# Grading meningioma resections: the Simpson classification and beyond

**DOI:** 10.1007/s00701-024-05910-9

**Published:** 2024-01-23

**Authors:** Matthias Simon, Konstantinos Gousias

**Affiliations:** 1 Department of Neurosurgery, Evangelisches Klinikum Bethel, Universitätsklinikum OWL, Bielefeld, Germany; 2 Department of Neurosurgery, St. Marien Academic Hospital Luenen, University of Muenster, Luenen, Germany; 3 Medical School, University of Nicosia, Nicosia, Cyprus; 4 Department of Neurosurgery, Athens Medical Center, Athens, Greece

**Keywords:** Meningioma, Meningioma surgery, Extent of resection, Simpson grading

## Abstract

Technological (and also methodological) advances in neurosurgery and neuroimaging have prompted a reappraisal of Simpson’s grading of the extent of meningioma resections. To the authors, the published evidence supports the tenets of this classification. Meningioma is an often surgically curable dura-based disease. An extent of meningioma resection classification needs to account for a clinically meaningful variation of the risk of recurrence depending on the aggressiveness of the management of the (dural) tumor origin.

Nevertheless, the 1957 Simpson classification undoubtedly suffers from many limitations. Important issues include substantial problems with the applicability of the grading paradigm in different locations. Most notably, tumor location and growth pattern often determine the eventual extent of resection, i.e., the Simpson grading does not reflect what is surgically achievable. Another very significant problem is the inherent subjectivity of relying on individual intraoperative assessments. Neuroimaging advances such as the use of somatostatin receptor PET scanning may help to overcome this central problem. Tumor malignancy and biology in general certainly influence the role of the extent of resection but may not need to be incorporated in an actual extent of resection grading scheme as long as one does not aim at developing a prognostic score. Finally, all attempts at grading meningioma resections use tumor recurrence as the endpoint. However, especially in view of radiosurgery/radiotherapy options, the clinical significance of recurrent tumor growth varies greatly between cases.

In summary, while the extent of resection certainly matters in meningioma surgery, grading resections remains controversial. Given the everyday clinical relevance of this issue, a multicenter prospective register or study effort is probably warranted (including a prominent focus on advanced neuroimaging).

## Introduction

**Fig. 1:**
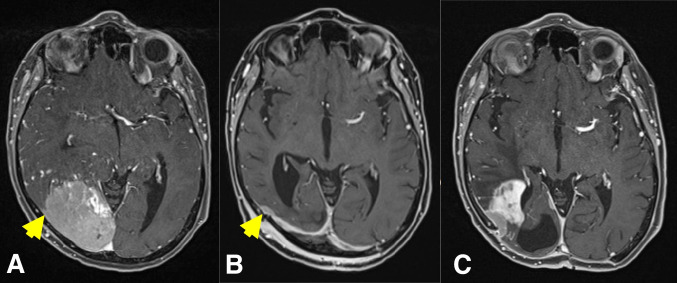
**A** September 2021: a large right occipital meningioma diagnosed in a 69-year-old woman (atypical meningioma WHO/CNS grade II). **B** December 2021: MR scans obtained 2 weeks after removal of an infected bone flap and epidural abscess. A small contrast-enhancing structure was interpreted as reactive tissue but in retrospect likely was residual tumor (yellow arrow). **C** October 2022: large recurrence likely originating from the small meningioma remnant

In September 2021, we removed a large right occipital meningioma in a 69-year-old woman who presented with slight personality changes (Fig. 1A). Intraoperatively, the tumor was found to originate exclusively from the occipital convexity dura which was generously excised and replaced with a synthetic dura substitute. There were no attachments to the sinuses, falx, or tentorium. There was also no apparent brain invasion, and a cleavage plane was easily developed using cottonoids. The tumor was very vascular, but there was no prominent cortical blood supply. The neuropathological work-up revealed an atypical meningioma WHO/CNS grade II. Based on the intraoperative impression of a complete resection (and an early postoperative CT scan), a 3-month follow-up MR scan was recommended.

Ten weeks after the surgery, the patient presented with an epidural abscess and had surgery for the removal of the bone flap. MR imaging in December 2021 showed a resolution of the infection (Fig. 1B); however, the patient did not come back to our department for further follow-up until October 2022. At that time, MR imaging revealed a large multifocal tumor recurrence (Fig. 1C). In retrospect, a possible tumor residual at the antero-inferior border of the bone flap was identified on the December 2021 scans from which the tumor regrowth may have originated, i.e., the tumor was likely never completely resected. The patient had repeat surgery and radiotherapy.

This case illustrates one of the central problems in meningioma surgery. Residual (dural) tumor matters and therefore also the degree of resection—at least in some patients. Of note, there is a surprisingly small body of data supporting long-term wait and scan policies in cases with residual meningioma after surgery [16, 24, 39]. Gillespie et al. have recently reported a series of 236 patients with “incomplete” meningioma surgeries, i.e., cases with tumor remnants described by the operating surgeon and/or depicted by postoperative MR. Using the RANO criteria, 56% of the cases showed radiological progression after a median follow-up of 64.4 months. Importantly, these authors did not confirm a prognostic impact of residual tumor volume and only to some degree (using the “local” radiological/tumorboard assessment but not the RANO criteria) the role of the histological grade. The Kaplan–Meier estimates of progression-free survival seemed to suggest that all tumors will eventually regrow [17].

## The Simpson grading

If the extent of a meningioma resection impacts the risk of tumor recurrence, then a classification thereof is needed. Meningiomas are believed to arise from meningeal (“arachnoid cap”) cells and therefore almost always involve the dural linings of the brain and spine. Recurrent tumors often originate from the dural margin of the initial resection (as in our case) [42, 43, 45]. From a surgical point of view, meningiomas are therefore dura-based growths with an intradural mass and sometimes extradural extensions. Hence, any classification of the extent of meningioma resections will have to account for how the dural infiltration has been dealt with. Probably, this line of reasoning and the attendant surgical observations which are usually detailed in the surgical notes led D. Simpson to propose a grading scheme for meningioma resections in his 1957 seminal article focusing on surgical management of the dural tumor infiltration which is still widely used today [54].

Briefly, Simpson distinguished between truly complete resections including the intradural part of the tumor, its dural attachment, and any extradural disease (grade I), surgeries which remove any macroscopic intra- and extradural disease but during which the dural origin of the tumor is coagulated rather than resected (grade II), complete resections of the intradural tumor leaving tumor infiltrated dura and extradural extensions behind (grade III), partial removal with residual intradural tumor (grade IV), and biopsies or decompressions, i.e., surgeries which do not aim at cytoreduction (grade V). He validated this grading scheme using clinical data from 288 cases. Recurrence rates were found to vary from 8.9% (grade I), 15.8% (grade II), 29.2% (grade III), to 46.7% (grades IV and V). The overall relation between the degree of resection and recurrence as well as the “dura-centric” assumption of prognostically important distinctions between Simpson resection grades I, II, and III has been widely accepted by the neurosurgical community in the past as one of the fundamental tenets of clinical meningioma management.

## Extent of resection matters: current relevance of the Simpson grading

However, the decades following Simpson’s publication have seen much technological progress, the advent of microsurgical techniques, and many surgical adjuncts, as well as the development of modern radiosurgical (and radiotherapeutical) treatment options. For example, current neuroimaging allows for a precise anatomical delineation of the tumor mass and for serial imaging follow-up for recurrence or growth of residual tumor. Modern meningioma treatment has changed dramatically from the times of Simpson’s 1957 paper. Patients are often diagnosed with smaller and asymptomatic tumors, and there is also an increasing recognition of quality-of-life issues and the importance of a proper balance between survival and oncology on the one hand and preservation of function on the other. Together, this has led to overall less aggressive surgical approaches and attitudes—and to a critical reappraisal of the Simpson classification and the role of extent of resection–recurrence relationship in general [3, 9, 10, 12, 20, 23, 41, 44, 46, 51, 58, 59, 62, 64].

We have been able to study recurrence-free survival and functional outcomes in overall 901 consecutive patients undergoing surgery for a primary meningioma (716 WHO grade I, 174 grade II, and 11 grade III). Our data basically confirmed that the degree of resection correlates with meningioma recurrence and that there are relevant prognostic differences between the resection categories described by Simpson including significantly different recurrence rates between cases undergoing Simpson grades I, II, and III resections. A Simpson grade II rather than grade I resection more than doubled the risk of recurrence at 10 years in our overall series (18.8% vs. 8.5%). These differences were much larger in cases with non-benign (i.e., WHO grades II and III) tumors but were still seen after dropping the latter patients from the analysis. Of note, we found no evidence that a lower Simpson grade (i.e., more aggressive resections) correlated independently with adverse functional outcomes [20].

Others have studied their institutional experience in a similar manner [3, 9, 12, 20, 23, 41, 44, 46, 58, 59, 62, 64]. Chotai and Schwartz have recently reviewed the pertinent 2010–2021 literature [10]. In part, based on the (relative) lack of statistical significance in several studies when comparing the recurrence rates between patients undergoing Simpson grades I, II, and III resections, Chotai and Schwartz argue that the Simpson classification of the extent of resection should be abandoned in favor of a grading scale that distinguishes primarily between “gross total” (i.e., Simpson grades I–III) and a partial resections (Simpson grades IV and V). We find it somewhat difficult to follow this line of reasoning. Twelve of 14 studies included in the Chotai and Schwartz paper reported the lowest recurrence rates in the Simpson grade I resection category. The risk of recurrence was found to increase step-by-step with the Simpson grade in 10 papers. Importantly, follow-up in virtually all pertinent studies is limited to a mean/median of ≤ 5 years only [3, 9, 10, 12, 20, 23, 41, 44, 46, 51, 58, 59, 62]. Only one of the papers included in the review by Chotai and Schwartz reports a median follow-up of 10 years [64]. However, at least in our series, the differences between the recurrence rates of patient subsets defined by the Simpson grade enlarged over time [20]. In summary, to the authors, the pooled evidence in the literature does suggest that there are prognostic differences between resection grades defined by different ways of dealing with the dural tumor origin.

**Fig. 2:**
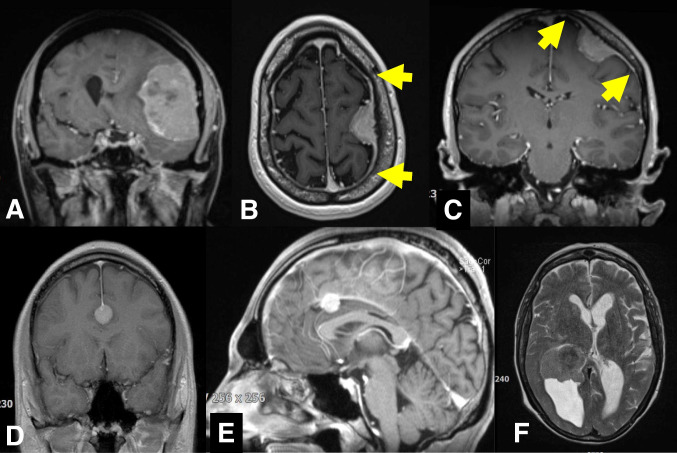
**A** and **B**, **C** Two cases with convexity meningiomas. Resecting the dural tumor origin of a convexity tumor is particularly easy. In the second case (**B**, **C**), there is a somewhat prominent “dural tail” (yellow arrows). At surgery, there was prominent dural hypervascularity but also a thin dural tumor layer surrounding the actual tumor mass. The latter observation together with the finding of microscopic dura infiltration [6, 7, 23] may argue for removal of “dural tails” in order to achieve a complete tumor resection. Of note (and as in our case), not all dural contrast enhancement depicted by MR scanning corresponds to actual tumor invasion (and not all invasion is detected by MRI). Incidence and extent of meningioma infiltration probably vary with the radiological characteristics of the “dural tail” [1, 47, 55]. **D**, **E** A small 15 mm meningioma originating from the lower border of the frontal falx. Simpson grade I resections of meningiomas of the falx are usually possible at little if any additional risk to the patient as long as there is some distance to the superior sagittal sinus. Infiltration of the superior sagittal sinus in falcine and convexity “parasagittal” meningiomas may preclude Simpson grade I, II and even III resections. It is however usually safely possible to resect the edge of the sinus, and one can follow the tumor into the sinus for removal of smaller intrasinusoidal fragments [18, 34]. **F** Intraventricular meningiomas have no true “dural” origin but rather originate from arachnoid cap cell populations in the choroidal plexus. The need to obtain hemostasis necessitates coagulation and removal of parts of the plexus, i.e., most of the times the surgery quite automatically results in a Simpson grade I resection

**Fig. 3:**
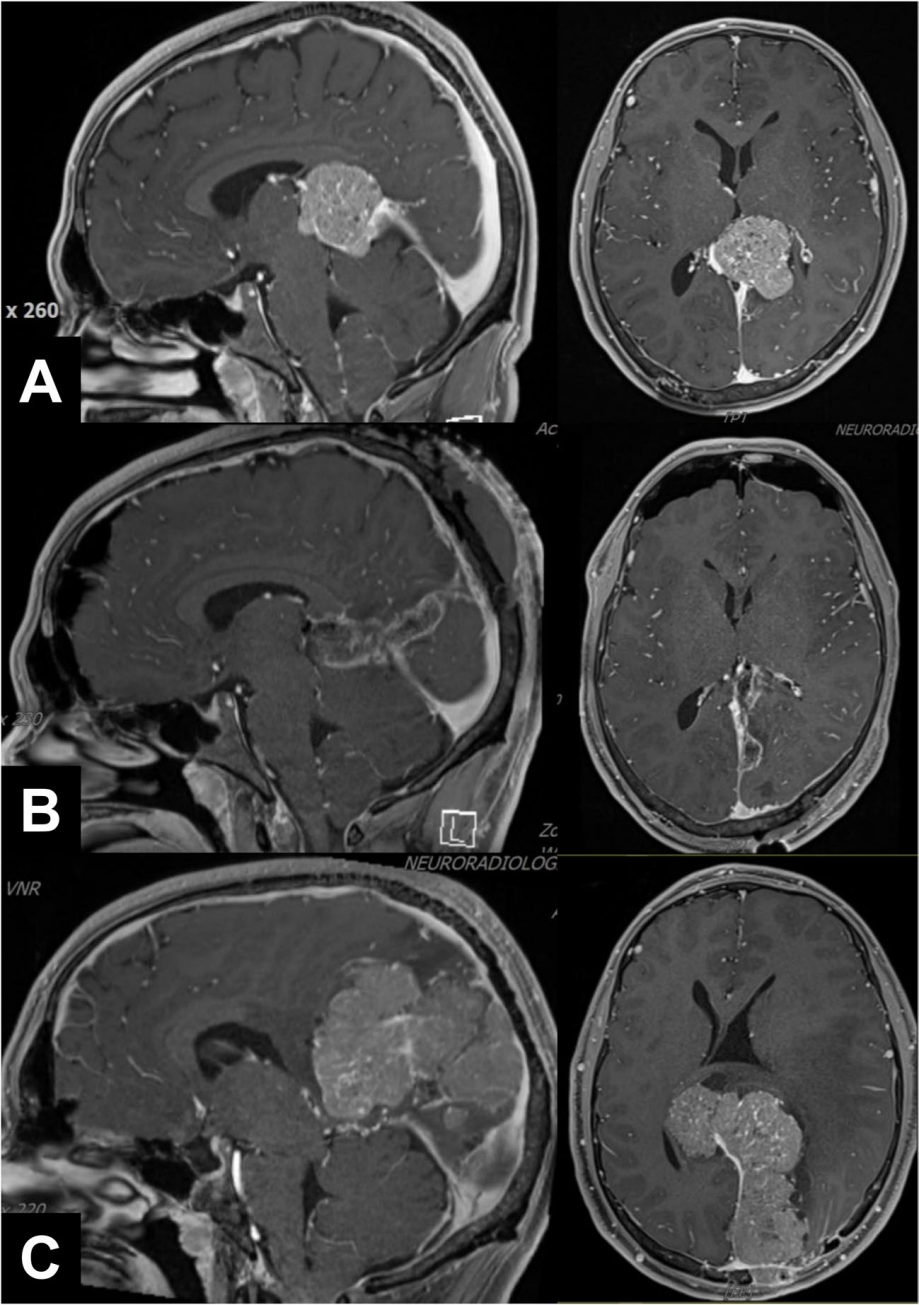
**A** Atypical falcotentorial meningioma WHO/CNS grade II operated following a first generalized seizure in a 20-year-old male. **B** Postoperative MR imaging confirmed the intraoperative impression of a Simpson grade III resection. **C** The patient did not report back to our center until 53 months later. At this time, there was a very large recurrence likely originating from the tumor infiltrated dura left behind during the initial surgery. However, resecting the dural tumor origin, i.e., the sinuses involved in this case during the first operation would not have been safe

**Fig. 4:**
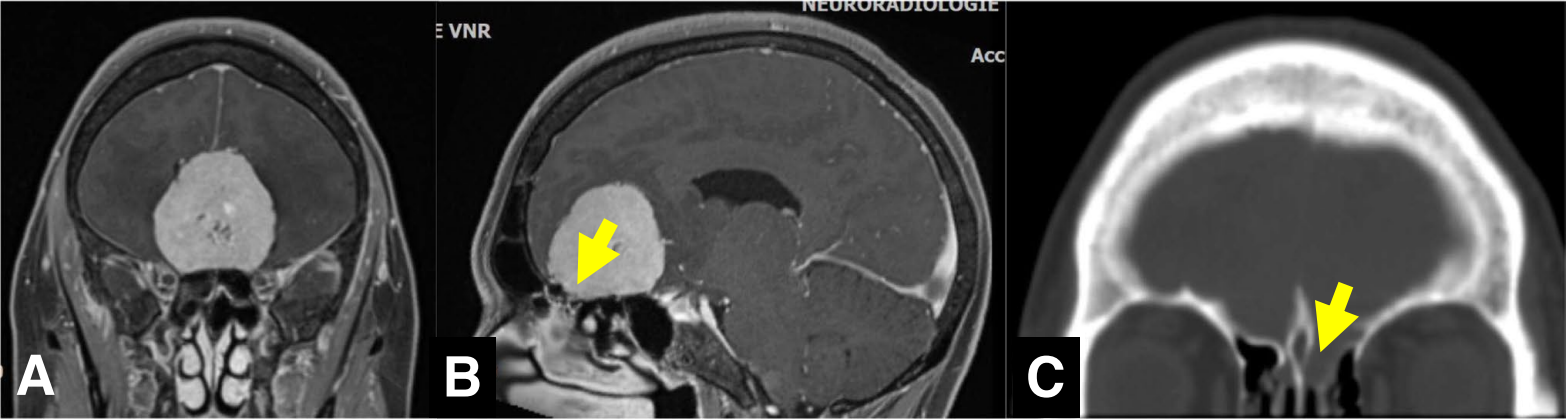
**A** A large olfactory groove meningioma with **B**, **C** infiltration of the bony skull base (yellow arrow). Resection of the tumor infiltrated dura and bone (Simpson grade I) may be a bit cumbersome and result in the need of reconstructing the skull base using, e.g., a periosteal flap, but does not add much risk to the operation

It should also be noted that the conceptual use of the Simpson grading scheme directly influences surgical strategies and decision-making beyond the mere prediction of recurrence rates. For example, are extensive dural resections necessary in convexity meningioma surgery (Fig. 2)? Should one resect and suture the edge of the sagittal sinus in parasagittal meningioma surgery? How aggressively should major sinus involvement be dealt with [18, 34] (Figs. 2 and 3)? What about the tentorium [52]? Is resecting (and possible grafting) the basal dura after removal, e.g., of an olfactory groove meningioma, appropriate and what are the implications for endoscopic surgery [5, 38] (Fig. 4)? Use of the Simpson grading implies that there are clinically relevant prognostic differences between Simpson grades I, II, and III, which in turn requires the surgeon to manage the dural origin of the tumor as aggressively as safely possible. On the contrary, a distinction between gross total and partial resections only essentially sends the message that meningioma surgery should merely aim at the removal of the intracranial tumor mass.

## Problems with grading the extent of meningioma resections

### Tumor location

**Fig. 5:**
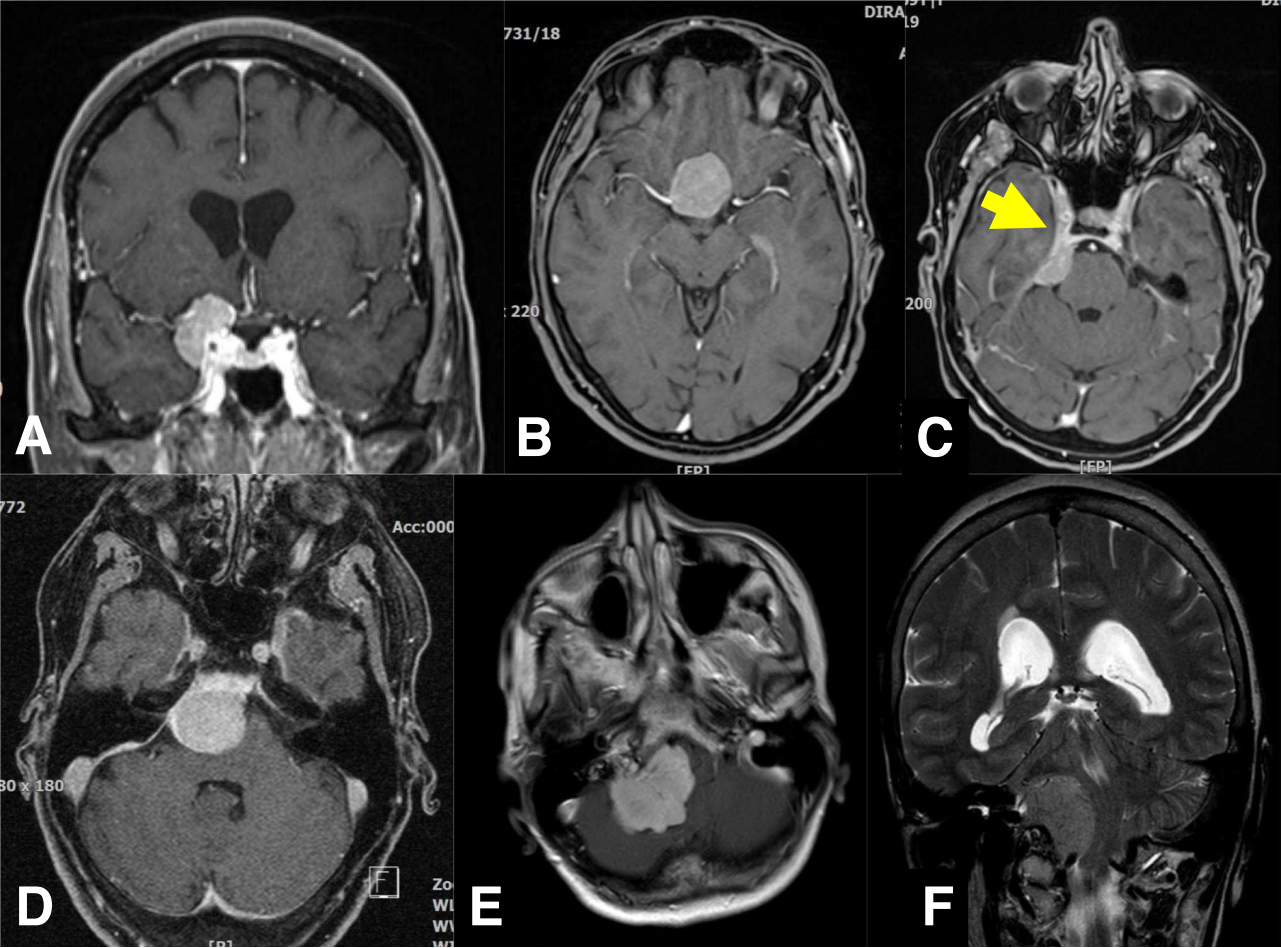
In most cases with skull base menigiomas, it may be technically very challenging to achieve even a Simpson grade III resection. **A** Depending on the specifics of cavernous sinus involvement the dural tumor origin of medial sphenoid wing and anterior clinoidal meningiomas can sometimes be resected. We do not routinely enter the cavernous sinus and at most remove its outer dural layer. **B** In cases with tuberculum sellae/planum sphenoidale meningiomas, tumor infiltration of the diaphragma sellae and the optic canals may limit the aggressiveness of the resection. **C** We usually do not resect the tentorial edge or enter the cavernous sinus (yellow arrow). **D** In clival and** E**, **F** petroclival meningiomas, resections are limited by the necessary manipulation of the brainstem, cranial nerves, and their vasculature. The cases shown had some coagulation of the dural tumor origins (i.e., Simpson grade II/III) resections

**Fig. 6:**
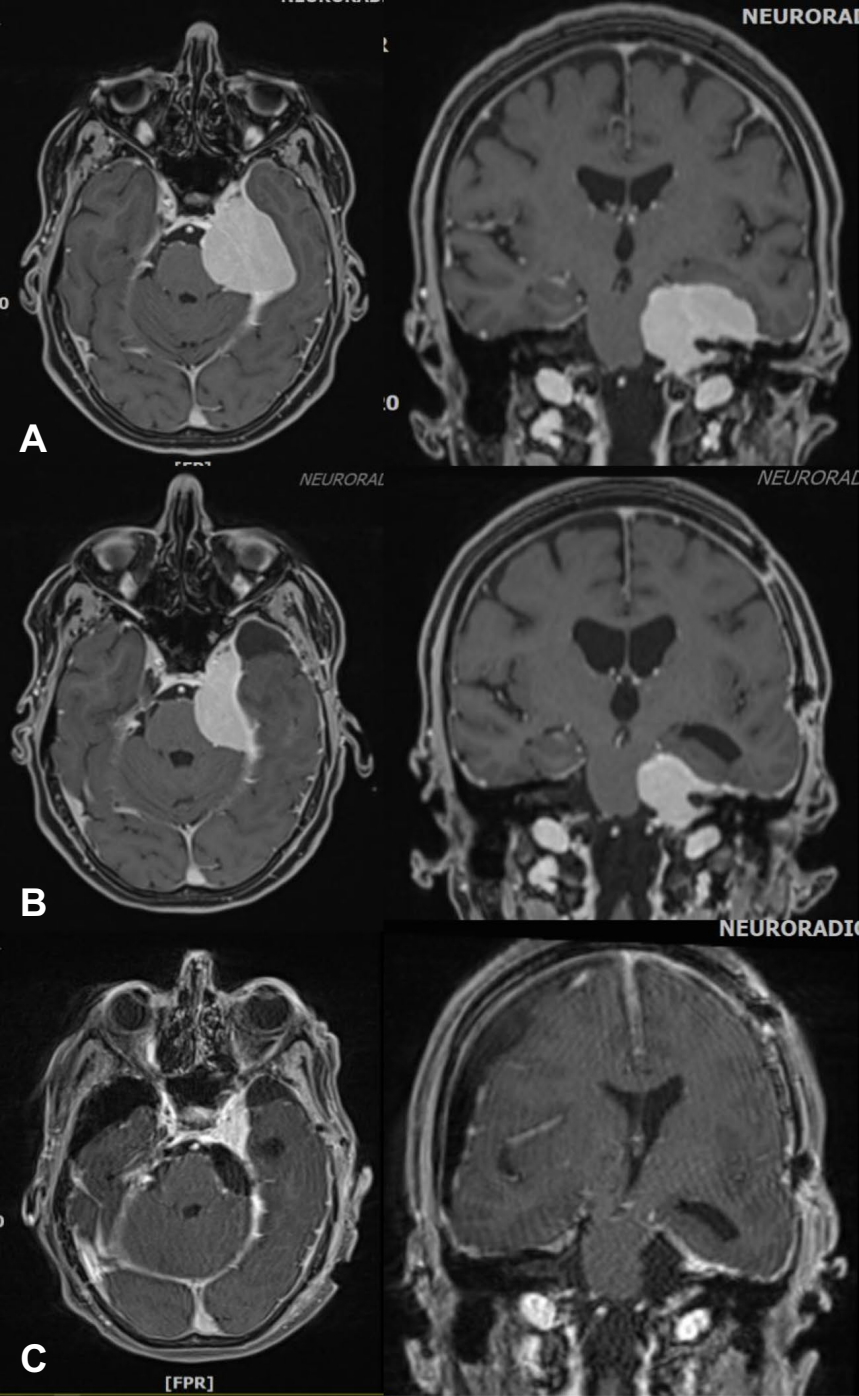
In some cases, it may be best to aim for an incomplete (staged) resection combined with later radiosurgery. **A** Large spheno-petro-clival meningioma diagnosed in a 46-year-old female presenting with a seizure. **B** The supratentorial part of the tumor was removed through a transsylvian-subtemporal route. **C** The patient had a second surgery 3 months later for resection of the infratentorial tumor using a lateral suboccipital route. Similar to others, we often prefer staged surgery and standard rather than true one-stage skull base (e.g., transpetrosal) approaches [15, 50]

**Fig. 7:**
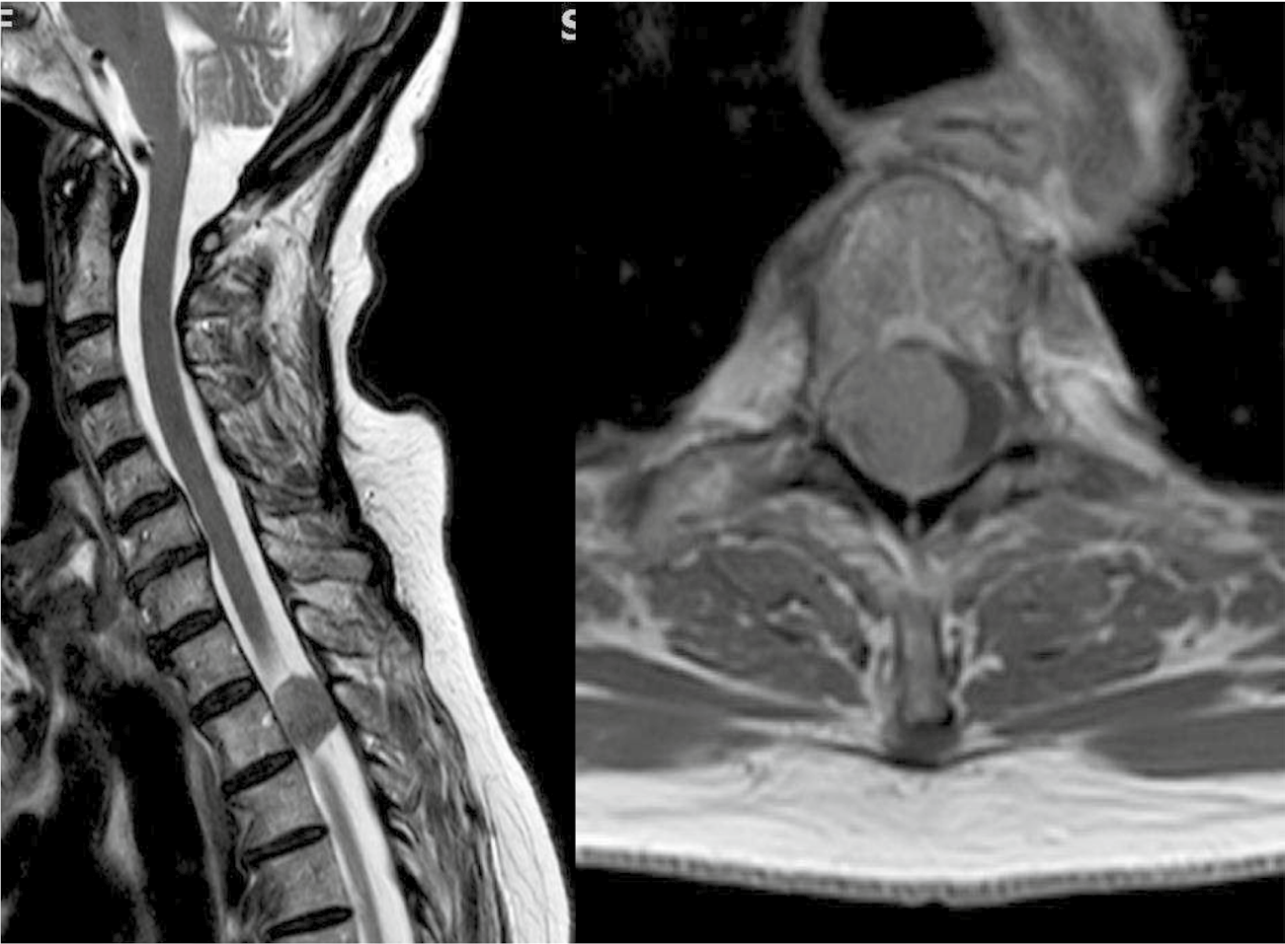
Spinal meningiomas most often originate from the antero-lateral dura. The authors remove the inner dural layer (“Simpson grade 1.5”) in such cases and try to avoid dural resections and reconstructions which require spinal cord traction and carry a significant risk of incurring CSF fistulas

The Simpson grading paradigm has not only been criticized based on analyses of published recurrence rates. Several other (and possibly more valid) concerns have also been expressed. First of all, tumor location is often the primary determinant of the eventual extent of resection [62]. For example, per routine convexity (and many falcine), meningiomas are completely excised together with the dural origin (Fig. 2) and even including a dural safety margin, i.e., only in a few cases the degree of resection will not correspond to Simpson grade I [23, 62]. Simpson grade I resections are also relatively easy to perform for some skull base tumors (Fig. 4). On the other hand, involvement of the major sinuses (Fig. 3) and encasement of or the need to manipulate the brainstem, its arteries, and cranial nerves may preclude even a gross total resection in cases with skull base meningiomas (Figs. 5, and 6) [15, 50]. Avoiding spinal cord traction and CSF fistulas are major issues in spinal meningioma surgery (Fig. 7). It would therefore appear that in many cases, the resection grades defined by the Simpson grading do not describe relevant and clinically useful categories.

In addition, the intraoperative assessment of the degree of resection which forms the basis of the Simpson grading scheme is inherently more difficult to apply in deep-seated tumors. This latter point has already been recognized by Simpson himself [54]. As a possible corollary of these issues, a recent analysis showed a differential prognostic impact of the Simpson grading in tumor subcohorts defined by tumor location. Specifically, a lesser influence of the extent of resection was seen in meningiomas of the skull base, posterior fossa, and falx. Some authors have therefore tried to modify the Simpson grading in order to better reflect the clinical realities, e.g., of skull base meningioma surgery [11, 40].

### Accounting for tumor malignancy, biology, and infiltration patterns

Alternatively, a differential impact of the Simpson grading in meningioma subsets might also in part reflect systematic pathobiological differences. Malignancy is one of the clinically most relevant aspects of meningioma tumor biology. The original Simpson grading was published at a time when histological grading was not applied to meningiomas, and the clinical cohort on which the classification was based likely included no or only a very few non-benign tumors. Indeed, some authors explicitly comment on the use of the Simpson classification in cases with benign tumors only [41, 44, 46, 59]. However, the histological tumor grade heavily influences recurrence rates. All studies focusing on atypical and malignant meningiomas describe a very substantial impact of the extent of resection on recurrence rates. Most use the Simpson classification grading in order to show very significant differences in recurrence-free survival following gross total (usually corresponding to Simpson grades I and II, but not III, vs. subtotal resections Simpson grades III–V) [8, 25, 33, 57]. Distinguishing between these latter two resection categories may at least in part reflect limited cohort sizes and the relatively low number of cases with grade IV (and V) surgeries. Some (but not all) authors describe differential recurrence rates after Simpson grades I and II resections [8, 20, 25]. Adjuvant radiotherapy is commonly prescribed in patients with non-benign meningiomas and most often in cases with residual tumor which constitutes a major confounder when investigating the influence of the extent of resection on recurrence-free survival in such cases [8, 25, 53, 57].

Of note, meningiomas also differ with respect to their infiltration pattern. Is the prognostic relevance of residual dural, osseous, extracranial, and/or brain invasion similar? The Simpson grading has been criticized for not properly addressing the specifics and nuances of the tissue infiltration pattern, and already, Simpson himself was a bit critical of the role of residual extracranial (and in particular bony) disease in tumor recurrence [54]. A current review of the literature attributes some importance to osseous infiltration in tumor regrowth; however, it appears to be quite difficult to dissect the impact of residual bony tumor from residual tumor in general and in particular tumor grade [60].

Another important issue is the problem of multifocal (dural) tumor growth. During meningioma surgery, one not uncommonly observes tumor satellites or a thin tumor layer in the vicinity of the main tumor mass (Fig. 2B, C), and microscopic tumor islets in the adjacent (convexity; other sites are obviously difficult to study) dura are a frequent finding if looked for by the neuropathologist. This phenomenon has been termed regional multifocality [6, 7]. Multifocality certainly calls into question the concept of a surgical cure for meningiomas [51]. On the other hand, these findings support one of the basic tenets of the Simpson classification, i.e., that meningioma is a dural disease and that therefore any classification of surgical meningioma cytoreduction will have to account for the dural origin of the tumor. Importantly, superior recurrence outcomes were reported by Kinjo et al. who routinely excised a 2 cm dural safety margin during convexity meningioma surgery. These authors referred to their practice as Simpson grade 0 resections [26].

A classification of the extent of resection for meningiomas may also have to account for brain invasion. Brain invasion is commonly seen together with features of cellular malignancy in higher-grade meningiomas (if looked for) [4], but brain invasion has been repeatedly discussed as a stand-alone criterion for more aggressive behavior and higher recurrence rates (brain invasive but otherwise benign meningiomas, BIOBM) [2, 21].

Beyond the malignancy and other issues, recent years have seen much overall progress in meningioma genetics and molecular biology in general [63]. It appears that not all meningiomas are the same, and future meningioma classifications may be need to based at least in part on molecular and genetic parameters [29, 48, 65]. Interestingly, meningioma molecular biology and genetics may well vary with tumor location [29, 48, 65]. If at some point in the future meningiomas are no longer meningiomas but are rather seen as different diseases not only from a basic science, but also from a clinical perspective, then the classification of the extent of resection of course may end up varying with tumor biology.

### Imaging and subjectivity issues

The Simpson grading is essentially a somewhat formalized way of stating an individual surgeon’s subjective post hoc assessment of an operation. Neuroimaging may conceptually overcome many of these problems. Slot et al. have recently proposed a classification scheme which relies on MR imaging in addition to intraoperative assessments and distinguishes between resections including the tumor origin, surgeries leaving the tumor origin behind, partial resections, and decompressions only [56]. Postoperative imaging can also help to better assess prognostic differences related to the amount of tumor left behind after partial resection. Intuitively, the size of the residual tumor should impact the risk of recurrence. Interestingly, there is already some evidence that an imaging-based and quantitative perspective on the role of meningioma cytoreduction evaluating residual tumor volume may be promising and may overcome some of the criticisms of the Simpson grading [9, 58]. Some authors have provided data suggesting a threshold between 3 and 5 cm^3^ in order to distinguish between two partial resection categories [13, 14, 32].

However, meningioma neuroimaging has to meet special challenges, i.e., the delineation of tumor not only in the brain but also in the dura, bone, and extracranial tissues. [68 Ga]-somatostatin receptor PET imaging (DOTATATE or DOTATOC PET) may help to better delineate tumor infiltration than more traditional MR scanning alone [22, 37, 61]. Some studies suggest that delineating radiotherapy targets based on a comprehensive neuroimaging work-up including PET scanning may have superior results, i.e., conventional MR imaging will sometimes underestimate the true extent of a meningioma. The recently published Copenhagen grading for meningioma resections attempts to overcome the subjectivity issue while preserving the idea of meningiomas as a localized and therefore surgically curable (dural) disease by combining neuropathological assessments of dural surgical margins with MR as well as [68 Ga]-DOTATOC PET imaging in order to assign resection grades [22, 35]. The somewhat laborious and costly intraoperative as well as imaging work-up may be justified by sparing many patients an extensive year-long imaging follow-up.

### The endpoint: tumor recurrence

Another important limitation of all extent of resection classifications is their focus on tumor recurrence as the eventual endpoint. Indeed, it is true that repeat surgery for meningiomas carries substantial risks which implies that avoiding tumor regrowth and recurrence is important for patients [27, 30, 31]. This is often overlooked in the discussion of the optimal extent of resection in a given patient since postponing risks (even at the price of larger risks in the future) might be quite attractive to both the patient and the neurosurgeon when faced with a difficult scenario.

However, not all recurrences are equally “bad.” At least, some cases with slowly progressive tumor can probably be followed safely. Conceptually, serial MR follow-up may avoid risky surgical interventions for large growths in elderly and comorbid patients in favor of earlier surgery or radiosurgery (and radiotherapy) [16, 19]. It is also possible to substitute (additional) radiosurgery/radiotherapy for aggressive microsurgery, i.e., in the cavernous sinus. Hybrid combined open and radiosurgery (Fig. 6) may be safer than aggressive surgery alone in some cases without compromising tumor control [19, 28, 49].

Finally, it should be noted that all relevant studies in the literature are retrospective and therefore often do not properly distinguish between symptomatic recurrences and mere imaging findings. From a somewhat strict methodological point of view, all current concepts of classifying the extent of meningioma resections with recurrence as the endpoint are based on limited-quality data.

## Conclusions and outlook

There seems to be general agreement that the saying “a lot helps a lot” applies to meningioma resections, i.e., there is a clinically relevant relationship between the extent of resection or residual tumor and the risk of tumor recurrence. Even the most outspoken critics of the Simpson grading do want to retain the overall concept that the extent of meningioma resection matters [10, 51]. Rather, much of the debate seems centered on how to define clinically useful resection categories.

More precisely, the relevance of distinguishing between Simpson grades I, II, and III resections is often called into question. To the authors, the current literature supports these distinctions [3, 9, 10, 12, 20, 23, 41, 44, 46, 51, 58, 59, 62, 64]. Recurrence rates seem to vary significantly between patient subsets undergoing Simpson grades I, II, and III resections. The problem of a relative lack of statistical significance in some studies and the statistically more robust findings when distinguishing between gross total (Simpson grades I–III) and partial resections may well reflect other than truly biological issues such as the subjectivity of the intraoperative assessment underlying the Simpson paradigm. Even if the impact of dural resections may be limited, this is still something that can be influenced by the operating surgeon. Use of the Simpson grading scheme addresses and offers concrete guidance with respect to an everyday clinical problem, i.e., how to deal with the dural tumor origin. An extent of resection grading paradigm should not only reflect prognostic considerations but also practical (surgical) issues.

However, it is also clear that the Simpson classification has severe shortcomings including the already mentioned reliance on an individual and subjective assessment of an operation by the operating surgeon. Imaging advances may play a very prominent role in overcoming this limitation. In particular, PET imaging shows much promise and may be able to depict dural tumor deposits [22, 37, 61]. Attempts such as the Copenhagen grading paradigm at integrating advanced imaging in the assessment of meningioma surgery should therefore definitely be pursued [22, 35].

Some other issues such as the problems related to tumor location can also be addressed without abandoning the basic tenets of the Simpson classification. It may make sense to modify the classification of the extent of resection according to tumor location, e.g., distinguish between cases with complete dural resections, those with complete removal of the tumor mass but without resection of the dural tumor origin, and “other” in most cases with convexity, parasagittal, and falcine meningiomas [56]. On the other hand, a two-tiered scheme distinguishing between gross total and partial resections with an additional distinction between a “large” or “small” tumor residual may indeed be more appropriate in certain skull base tumors in which the resection of the dural tumor origin is not feasible [10, 15, 50, 51]. Of note, these modifications basically retain the original Simpson paradigm with its attendant focus on the dural tumor origin and pool or subdivide its categories.

Finally, even though the prognostic impact of the extent of resection may vary between patient groups, e.g., defined by malignancy grades or other aspects of tumor biology, this may not necessarily require the inclusion of such parameters in the grading scheme itself or (completely) different scales for different meningioma subsets. It may not be practical to attempt to develop a degree of resection grading into a prognostic score. The same argument may also have some value when thinking about how the undoubtedly important role of radiosurgical and radiotherapeutical treatment options can be accounted for.

Given the significant and controversial debate surrounding the classification of the extent of meningioma resections which has not succeeded in replacing an at the time of this writing 66 years old paradigm with something novel that everybody agrees on, it may be worthwhile to join forces and conduct a multicentric prospective study or establish a register. Such efforts could also help with another issue that for practical reasons is often not quite appropriately acknowledged: the clinical impact of meningioma recurrence varies vastly with size, growth dynamics, and its management. We certainly need a better endpoint for the assessment of our resection strategies and treatment concepts in general.

## Data Availability

NA.
